# The Relationship between Skin Symptoms and Allergic Reactions to Asian Dust

**DOI:** 10.3390/ijerph9124606

**Published:** 2012-12-10

**Authors:** Shinji Otani, Kazunari Onishi, Haosheng Mu, Yae Yokoyama, Takenobu Hosoda, Mikizo Okamoto, Youichi Kurozawa

**Affiliations:** 1 Arid Land Research Center, Tottori University, Tottori 680-0001, Japan; 2 Division of Surgical Oncology, Faculty of Medicine, Tottori University, Yonago 683-8504, Japan; 3 Japan Environment & Children’s Study, The Center of Tottori Unit of the JECS, Faculty of Medicine, Tottori University, Yonago 683-8503, Japan; E-Mail: issey@med.tottori-u.ac.jp; 4 Division of Health Administration and Promotion, Faculty of Medicine, Tottori University, Yonago 683-8503, Japan; E-Mails: muhs@med.tottori-u.ac.jp (H.M.); yaeyae@ns.cygnus.ac.jp (Y.Y.); thosoda@med.tottori-u.ac.jp (T.H.); mokamoto@med.tottori-u.ac.jp (M.O.); kurozawa@med.tottori-u.ac.jp (Y.K.)

**Keywords:** Asian dust, metal allergy, skin symptom, air pollutant, nickel

## Abstract

Asian dust events result from displacement of atmospheric pollutants from the Chinese and Mongolian deserts, causing associated health issues throughout Northeast Asia. We investigated the relationship between skin symptoms in Asian dust events and contact allergy to Asian dust and associated metals. Increases in atmospheric levels of heavy metals such as Ni, Al, and Fe occurred during the severe Asian dust event on March 21, 2010. We conducted a case–control study (n = 62) with patch testing to compare skin symptoms on an Asian dust day with metal allergic reactions. Skin symptoms were observed in 18/62 subjects. Nine subjects with skin symptoms (group A) and 11 without (group B) were patch tested for six metals and Asian dust particles. Metal and dust samples were applied to the subjects’ backs for 2 days and the reactions were scored according to the International Contact Dermatitis Research Group guidelines. Differences in the positive rates between the groups were analyzed. Skin reactions to ferric chloride (*p* = 0.015), aluminum chloride (*p* = 0.047), nickel sulfate (*p* = 0.008), and Asian dust particles (*p* = 0.047) were more common in group A than in group B. Skin symptoms during Asian dust events may be allergic reactions to Asian dust particle-bound metals.

## 1. Introduction

Asian dust events result from the long-range displacement of atmospheric pollutants originating in the Chinese and Mongolian deserts. Mineral or soil particles blown into the atmosphere are carried by westerly winds and reach Northeast Asia. Although Asian dust event frequencies and atmospheric dust levels have been increasing steadily in the eastern Asia region [1], the effects of Asian dust on human health are not well known. Recent epidemiological studies have shown that Asian dust storm events coincided with increases in daily admissions and clinical visits for allergic diseases such as asthma [2,3,4], allergic rhinitis [5], and conjunctivitis [6]. We previously reported the association between skin symptoms and Asian dust events [7] and found an association between levels of suspended particulate matter (SPM) and contaminating metals such as nickel [8].

Metals are common contact allergens, and nickel especially is a common cause of contact allergies in the general population [9]. Moreover, dermatologists and occupational physicians have become increasingly aware of airborne sources of contact dermatitis, which results mainly from exposure to irritants or allergens [10]. Nickel is a cause of airborne dermatitis, and inhaled nickel in ambient air has been suggested as a risk factor for nickel sensitization [11]. Patch tests are very easy and useful methods for estimation of contact dermatitis. We conducted a case-control study and used patch tests to compare skin symptoms on an Asian dust day with metal allergic reactions.

## 2. Experimental Section

### 2.1. Health Survey on the Effects of Asian Dust Events

We conducted a health survey on the effects of Asian dust events on daily subjective symptoms, including skin symptoms (itching, eczema, pain, and reddish skin); the survey period was from February to April 2010. The study subjects were 62 volunteers. They were recruited from two public agencies in Yonago, Japan after obtaining informed consent. They included 27 men and 35 women, with a mean (±SD) age of 39.8 ± 13.2 years. The subjects had desk jobs and spent the better part of their days indoors and were without moderate or severe underlying disease (including severe allergic diseases). They were distributed survey sheets for skin symptoms. Each symptom was defined by self-checking visual analogue scale, and the subjects checked their symptoms on the survey sheets every evening. The severities of these symptoms were quantified are follows: 0 point, no symptom; 1 point, slight involvement; 2 points, mild involvement; 3 points, moderate involvement; 4 points, severe involvement; and 5 points, extreme involvement. This study was reviewed and approved by the Ethics Committee of the Faculty of Medicine, Tottori University.

### 2.2. Asian Dust Day Data

In total, five Asian dust days (visibility <10 km, as determined by the Japan Meteorological Agency) were identified during the survey period. One of these was a severe Asian dust event (visibility <3 km) observed on 21 March 2010. The daily average hourly levels of SPM and atmospheric heavy metals (Pb, Cr, Mn, Cd, Ni, Cu, Zn, Fe, Ca, and Al) in a 3-day period beginning before and ending after the Asian dust day in Yonago were measured by inductively coupled plasma-atomic emission spectroscopy (Model SPS3520UV; SII NanoTechnology, Chiba, Japan). These data were obtained from Tottori Prefectural Institute of Public Health and Environmental Science. We counted out other 4 Asian dust days; March 13 and 16, and April 29 and 30, because of not so high levels of SPM; 28 µg/m^3^, 39 µg/m^3^, 19 µg/m^3^, and 27 µg/m^3^in the daily averages for the hourly levels of SPM, respectively.

### 2.3. Sampling and Measurement of Metal Elements in Total Suspended Particulates

TSPs were obtained by evaporation after collection in a simple flower bowl 798 mm in diameter and 300 mm deep at Tottori Prefectural Institute of Public Health and Environmental Science from March 19 to 23, 2010. Ten elements (Pb, Cr, Mn, Cd, Ni, Cu, Zn, Fe, Ca, and Al) from the corrected TSPs were measured by inductively coupled plasma–mass spectrometry (Model 7500i, Agilent Technology, USA). These data were obtained from Murata Keisokuki Service Co., Ltd., Yokohama, Japan. The patch test dust samples were prepared as 1 mg corrected TSPs in 100 mg Vaseline^®^.

### 2.4. Case-Control Study with Patch Tests

**Figure 1:**
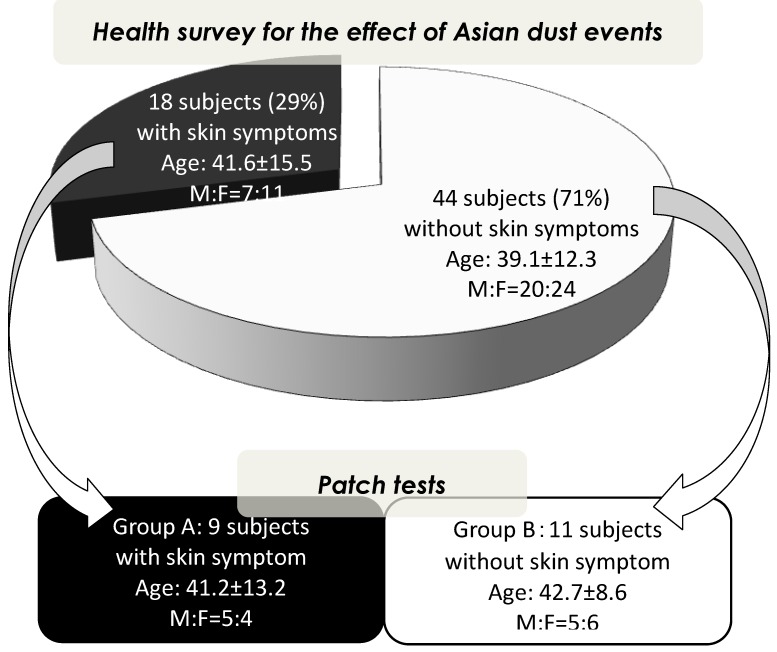
Scheme of case-control study.

As presented in Figure 1, on the Asian dust day in this study (March 21, 2010), 18 subjects (seven men and 11 women, mean age of 41.6 ± 15.5 years) had skin symptoms and 44 (20 men and 24 women, mean age of 39.1 ± 12.3 years) had no skin symptoms. All subjects have not been sensitized by other allergens around the Asian dust day. We obtained permission to perform patch testing from 20 subjects: nine with skin symptoms (group A: five men and four women; mean age, 41.2 ± 13.2 years) and 11 without skin symptoms (group B: five men and six women; mean age, 42.7 ± 8.6 years) on the Asian dust day. Between the group with skin symptoms, the group without skin symptoms, group A, and group B, there are no differences in age and sex (*p* = 0.798 and *p* = 0.884).

We prepared six commercially available metals for use in the patch tests: Zn (2% ZnCl_2_), Mn (2% MnCl_2_), Cr [2% Cr2(SO4)3], Fe (2% FeCl_3_), Al (2% AlCl_3_), and Ni (5% NiSO_4_) (Torii Pharmaceutical, Tokyo, Japan). Patch tests that included the 6 metals described above and a dust sample were applied to the subjects’ backs for 2 days, with vinyl plaster (Patch Tester Torii; Torii Pharmaceutical, Tokyo, Japan). The skin symptoms were graded according to the International Contact Dermatitis Research Group scoring system 3 days after application. For the patients who had doubtful or vague reactions 3 days after the sample applications, another reading was made on day 7. Reactions of + to +++ were regarded as positive. Reactions of − and ± were negative and doubtful, respectively.

### 2.5. Statistical Analysis

The chi-square test and Fisher’s exact test (in case of one or more of cells having an expected frequency of five or less) were used to analyze differences in the positive rates between groups A and B. One-way analyses of variance was used for intergroup differences in age and sex. The level of statistical significance was set at 5%. SPSS 19.0 for Windows (IBM SPSS Inc., Chicago, IL, USA) was used to perform all data analyses.

## 3. Results and Discussion

### 3.1. Results

**Figure 2:**
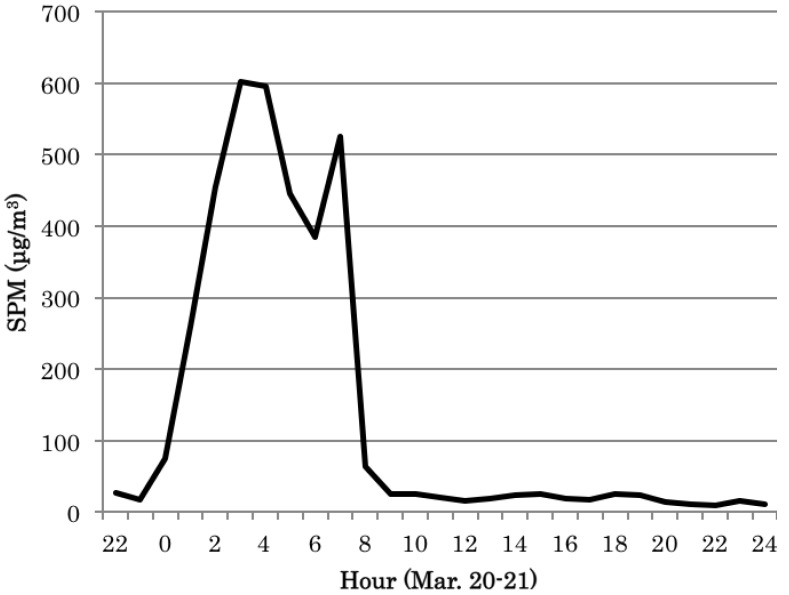
Time series of hourly SPM levels from the 20th to 21st of March 2010.

The daily averages for the hourly and maximal hourly levels of SPM (particles with <10-µm diameter) on the target Asian dust day were 151 µg/m^3^ and 602 µg/m^3^, respectively (environment quality standard in Japan: the daily averages for the hourly and maximal hourly levels to not exceed 100 µg/m^3^ and 200 µg/m^3^, respectively). Figure 2 shows the time series of hourly SPM levels on the Asian dust event day. The levels of 10 atmospheric heavy metals were also increased on this Asian dust day (Table 1). The levels of all heavy metals on March 20 were higher than those on the days before and after. The levels of the atmospheric 10 metals in the corrected total suspended particulates (TSPs: all atmospheric particles) from March 19 to March 23, 2010 were shown Table 1.

**Table 1:** Levels of 10 atmospheric heavy metals (Pb, Cr, Mn, Cd, Ni, Cu, Zn, Fe, Ca, and Al) during the 3 days starting before and ending after the Asian dust day on March 21, 2010 (above), and levels of the atmospheric 10 metals in the corrected total suspended particulates (below).

**The levels of the atmospheric metals**										
**Date**	**Pb (ng/m^3^)**	**Cr (ng/m^3^)**	**Mn (ng/m^3^)**	**Cd (ng/m^3^)**	**Ni (ng/m^3^)**	**Cu (ng/m^3^)**	**Zn (ng/m^3^)**	**Fe (μg/m^3^)**	**Ca (μg/m^3^)**	**Al (μg/m^3^)**
March 20	26.2	6.0	39.4	0.9	4.3	10.0	74.6	1.3	1.7	1.0
March 21	57.2	46.0	584.9	1.9	33.3	36.2	199.5	15.2	13.6	9.3
March 22	10.8	2.3	14.4	0.2	1.2	2.8	29.1	0.5	0.7	0.5
**The levels of the atmospheric metals in the corrected total suspended particulates**										
**Date**	**Pb (mg/kg)**	**Cr (mg/kg)**	**Mn (mg/kg)**	**Cd( mg/kg)**	**Ni (mg/kg)**	**Cu (mg/kg)**	**Zn (mg/kg)**	**Fe (g/kg)**	**Ca (g/kg)**	**Al (g/kg)**
March 19 to 23	102	96	960	3	51	62	505	38	34	63

On the target Asian dust day (21 March 2010), 18 subjects of all participants (62 subjects) in the health survey had skin symptoms. The number of subjects with each skin symptoms and average points per person are as below: itching: 18 subjects, 1.4 points; eczema: 4 subjects, 0.4 point; pain: 3 subjects, 0.2 point; and reddish skin: 4 subjects, 0.3 point.

**Figure 3:**
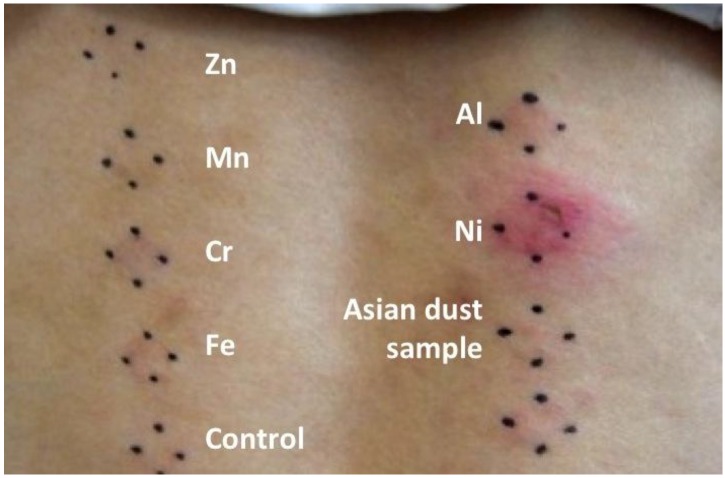
Photograph of the back of a subject in group A who positively reacted to the Cr (+), Fe (+), Al (+), Ni (+++), and Asian dust sample preparations (+) from the patch test.

Table 2 shows the results of the patch tests and the percentages of each reaction (+++, ++, +, ±, and −). Compared with group B, significantly more subjects in group A reacted to three metal sample preparations (Fe, *p* = 0.015; Al, *p* = 0.047; Ni, *p* = 0.008) and the Asian dust sample preparation (*p* = 0.047). Figure 3 is a photograph of the back of a subject in group A who showed positive reaction to Ni and Asian dust sample preparation.

**Table 2:** The results of the patch tests andthe percentages of each reaction. Chi-square test (*Fisher’s exact test) with a significance level of 5%. Compared with group B, significantly more subjects in group A reacted to three metal sample preparations (Fe, Al, and Ni) and the Asian dust sample preparation (labeled as D on the y-axis).

Sample	Group	+++	++	+	±	-	*p* value
Zn *	A	0 (0%)	0 (0%)	1 (11%)	1 (11%)	7 (78%)	0.189
	B	0 (0%)	0 (0%)	0 (0%)	0 (0%)	11 (100%)	
Mn *	A	0 (0%)	0 (0%)	0 (0%)	0 (0%)	9 (100%)	1.000
	B	0 (0%)	0 (0%)	0 (0%)	1 (9%)	10 (91%)	
Cr	A	0 (0%)	1 (11%)	2 (22%)	1 (11%)	5 (55%)	0.279
	B	0 (0%)	0 (0%)	1 (9%)	0 (0%)	10 (91%)	
Fe	A	0 (0%)	2 (22%)	3 (33%)	1 (11%)	3 (33%)	0.015
	B	0 (0%)	0 (0%)	0 (0%)	0 (0%)	11 (100%)	
Al	A	0 (0%)	0 (0%)	3 (33%)	1 (11%)	5 (55%)	0.047
	B	0 (0%)	0 (0%)	0 (0%)	0 (0%)	11 (100%)	
Ni	A	2 (22%)	2 (22%)	1 (11%)	3 (33%)	1 (11%)	0.008
	B	0 (0%)	0 (0%)	2 (18%)	0 (0%)	9 (82%)	
D	A	0 (0%)	0 (0%)	2 (22%)	2 (22%)	5 (55%)	0.047
	B	0 (0%)	0 (0%)	0 (0%)	0 (0%)	11 (100%)	

### 3.2. Discussion

The present study’s results indicate that subjects with contact allergy to metals may often be affected by Asian dust and show skin symptoms. In addition, our results suggested that skin symptoms associated with Asian dust events may be an allergic reaction to metals bound to Asian dust particles. To the best of our knowledge, this is the first report that has shown a direct association between Asian dust exposure and allergic reactions. We previously reported associations between skin symptoms and Asian dust events [7]. Japanese cedar pollen increases from around the same time as occurrence of Asian dust events in Japan. Although Japanese cedar pollinosis is a common disease that poses a major public health problem in Japan, health effects by pollen are mainly nasopharyngeal and ocular symptoms. In our previous study, skin symptom was significantly associated with the levels of SPM compared to the levels of pollen [8]. Moreover, there are no major sources of air pollution apart from a paper mill and motor vehicles in our research area. Therefore it is concluded that the skin symptoms were related to Asian dust exposure.

Particulates in Asian dust are principally composed of rock-forming minerals and clay minerals, such as quartz, feldspar, mica, kaolinite, and chlorite. Analysis of Asian dust particles has shown the presence of ammonium ions, sulfate ions, nitrate ions, and heavy metal compounds that are not considered to originate from the soil. Asian dust particles have been thought to adsorb anthropogenic atmospheric pollutants during transport [12].

The mechanism of worsening symptoms in case of asthma by Asian dust may be attributed to particulate matter [13]. Particulate matter is known to increase airway inflammation in asthma patients. The aggravated allergic inflammation may be because of certain mineral elements such as SiO_2_, which is the main component of Asian dust particles [14]. In case of skin symptoms, it is uncertain that the skin has the same mode of action as airway. A likely explanation is considered that skin symptoms result from airborne contact dermatitis. Airborne contact dermatitis is defined by the presence of the environment of dust, droplets, or volatile causative agents; the clinical symptoms; the history of the patient; and the results of epicutaneous tests [10]. Clinical findings of airborne contact dermatitis are similar to common contact dermatitis symptoms, and type 1, 3 and 4 hypersensitivity is incriminated as a cause of this type of dermatitis [15]. Most of the allergens of identified are in an occupational setting, and nickel is one of causes of airborne contact dermatitis [10]. In current study, it was unlikely to have a significant impact on the skin condition, because most of the subjects with skin symptoms had only itching and the low level of severities points on the Asian dust day. However, nickel associated Asian dust may have some influence on skin symptoms from results of the patch tests. The current study supports the idea that levels of Ni on Asian dust days may be significantly associated with skin symptoms [8].

The limitations of our study include the use of environmental data as a surrogate for actual exposure. Most of subjects were commuting to work around 8 am, and might be exposed to the Asian dust. However, the duration of exposure was short and subjects spent the better part of their days indoors. We need to evaluate a number of Asian dust events and collect more data, because we surveyed the only one Asian dust event and a small number of subjects in this study. Secondly, the interpretation of the patch test results for the Asian dust sample preparation may be overestimated. The particle diameters that reach Japan range up to a maximum of approximately 4 µm, and therefore, it is unlikely that skin reaction is caused by physical irritation of the Asian dust particle. Moreover, we did not investigate organic agents, such as fungi and bacteria, which may adhere to Asian dust particles [16,17,18]. Furthermore, the effect of Asian dust particles on clinical conditions other than skin symptoms is unclear. Thus, further studies are needed to evaluate the association of organic compounds in Asian dust particles and human health.

## 4. Conclusions

The present study demonstrates that subjects with skin symptoms on Asian dust day had a tendency toward metal allergy. In addition, our results suggested that skin symptoms associated with Asian dust events may be an allergic reaction to metals bound to Asian dust particles.
